# Standardizing Protein Corona Characterization in Nanomedicine: A Multicenter Study to Enhance Reproducibility and Data Homogeneity

**DOI:** 10.1021/acs.nanolett.4c02076

**Published:** 2024-08-03

**Authors:** Ali Akbar Ashkarran, Hassan Gharibi, Seyed Majed Modaresi, Amir Ata Saei, Morteza Mahmoudi

**Affiliations:** † Department of Radiology and Precision Health Program, Michigan State University, East Lansing, Michigan 48824, United States; ‡ Division of Physiological Chemistry I, Department of Medical Biochemistry and Biophysics, Karolinska Institutet, Stockholm 171 77, Sweden; § Biozentrum, University of Basel, 4056 Basel, Switzerland; ∥ Department of Microbiology, Tumor and Cell Biology, Karolinska Institutet, Stockholm 171 65, Sweden

**Keywords:** proteomics, mass spectrometry, protein corona, consistency, robustness, nanomedicine

## Abstract

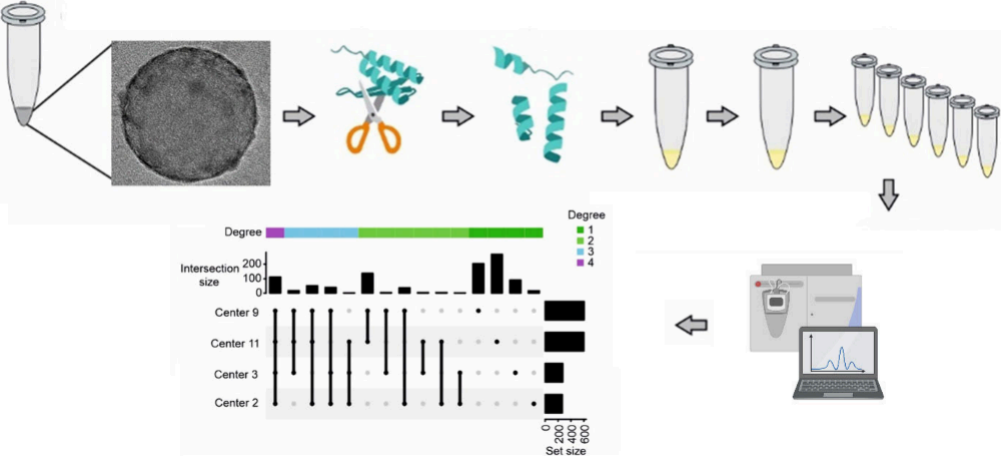

We recently revealed significant variability in protein corona characterization across various proteomics facilities, indicating that data sets are not comparable between independent studies. This heterogeneity mainly arises from differences in sample preparation protocols, mass spectrometry workflows, and raw data processing. To address this issue, we developed standardized protocols and unified sample preparation workflows, distributing uniform protein corona digests to several top-performing proteomics centers from our previous study. We also examined the influence of using similar mass spectrometry instruments on data homogeneity and standardized database search parameters and data processing workflows. Our findings reveal a remarkable stepwise improvement in protein corona data uniformity, increasing overlaps in protein identification from 11% to 40% across facilities using similar instruments and through a uniform database search. We identify the key parameters behind data heterogeneity and provide recommendations for designing experiments. Our findings should significantly advance the robustness of protein corona analysis for diagnostic and therapeutics applications.

The protein/biomolecular corona is a dynamic biological layer that develops on the surface of nanoparticles when they are exposed to biological fluids including plasma.^1−4^ This biological layer transforms nanoparticles by endowing them with a new identity that influences how they are recognized and interact within biological systems, thus defining their safety, biodistribution, diagnostic and therapeutic efficacy.^3,5,6^ Despite significant advances in nanomedicine, a limited understanding of nanoparticles’ biological identity remains a major barrier to their successful clinical translation.^7^ A profound comprehension of the protein corona composition is crucial for predicting their *in vivo* biological fate, which is essential for their clinical application and the advancement of future nanomedicine technologies.^7^

Over the past decade, extensive research has aimed to enhance the reproducibility of nanobio interfaces.^8,9^ Yet, the critical role of liquid chromatography mass spectrometry (LC-MS) in ensuring the robustness and reproducibility of protein corona data—a pivotal method for identifying and quantifying protein corona composition^10−12^—has received insufficient attention.^10−13^ Our recent studies show significant discrepancies in identical protein corona outcomes and interpretations across various proteomics facilities, attributed to differences in sample preparation protocols, LC-MS workflows, instrumentation, and data processing.^13^ Consequently, there is an urgent need to develop a standardized protocol for protein corona analysis to reduce data heterogeneity and facilitate comparability across studies.^13,14^

Our previous research indicates that major disparities in the characterization of the protein corona composition are primarily due to variations in sample preparation protocols, LC-MS workflows, and data processing.^13^ We also revealed that harmonizing database search and data processing can significantly reduce the observed heterogeneity among core facilities.^15^ Different proteomics core facilities employ a range of sample preparation methods, instrumentation, quantification approaches, search parameters, and data processing techniques. These differences can significantly bias the outcomes of the protein corona analyses of identical samples. Furthermore, the multistep nature of sample preparation—which includes protein denaturation, reduction, alkylation, digestion, and desalting/cleaning—introduces additional variability due to the diverse chemicals and reagents used across different laboratories.^16−18^ Additionally, the use of various LC and MS systems contributes to another layer of variation and heterogeneity that affects the final characterization of the protein corona.^19−21^

The primary goal of this follow-up study is to minimize the main sources of variations and improve the protein corona data homogeneity, focusing on harmonizing sample preparation protocols, LC-MS instrumentation and workflows, database searches, and processing strategies. Ultimately, similar to other nanomedicine techniques and methods,^6^ we aim to establish a standardized protocol that will streamline the analysis of the protein corona across different core facilities, facilitating the comparability of protein corona data sets from various studies. More specifically, here, we prepared identical ready-to-inject batches of digested protein corona samples and sent them to various proteomics centers for analysis. We used an identical on-bead digestion protocol for all protein corona samples and submitted the final 4 (out of 6) identical batches of dried peptides to 4 proteomics core facilities in the United States that had provided relatively higher quality data in on our previous study (called good centers) based on protein and peptide counts, coefficients of variation (CV) of technical replicates, and the median sequence coverage.^13^ In addition, to probe the role of LC-MS instrument in heterogeneity of protein corona data, we also sent another 4 identical batches of dried peptides to 4 proteomics core facilities that had similar instrumentation and were expected to perform similarly (i.e., identical LC and similar MS types; called similar centers). Two of these centers were shared with the above (good) centers, so in total we sent identical samples to 6 core facilities. Finally, we also performed a uniform database search on the raw data retrieved from the 4 centers with similar instruments, where the sample preparation protocol, instrumentation, and data processing would all be streamlined. In summary, we conducted the study in 3 steps: 1) unifying the sample preparation protocol; 2) unifying sample preparation protocol and the instrumentation used, and 3) unifying sample preparation protocol, instrumentation, and data processing. We show that implementation of each step reduces data heterogeneity of the protein corona composition across core facilities, which can be used for analysis of protein corona in the future, enabling rapid developments in nanomedicine-based diagnostics and therapeutics.

## Results

**Figure 1:**
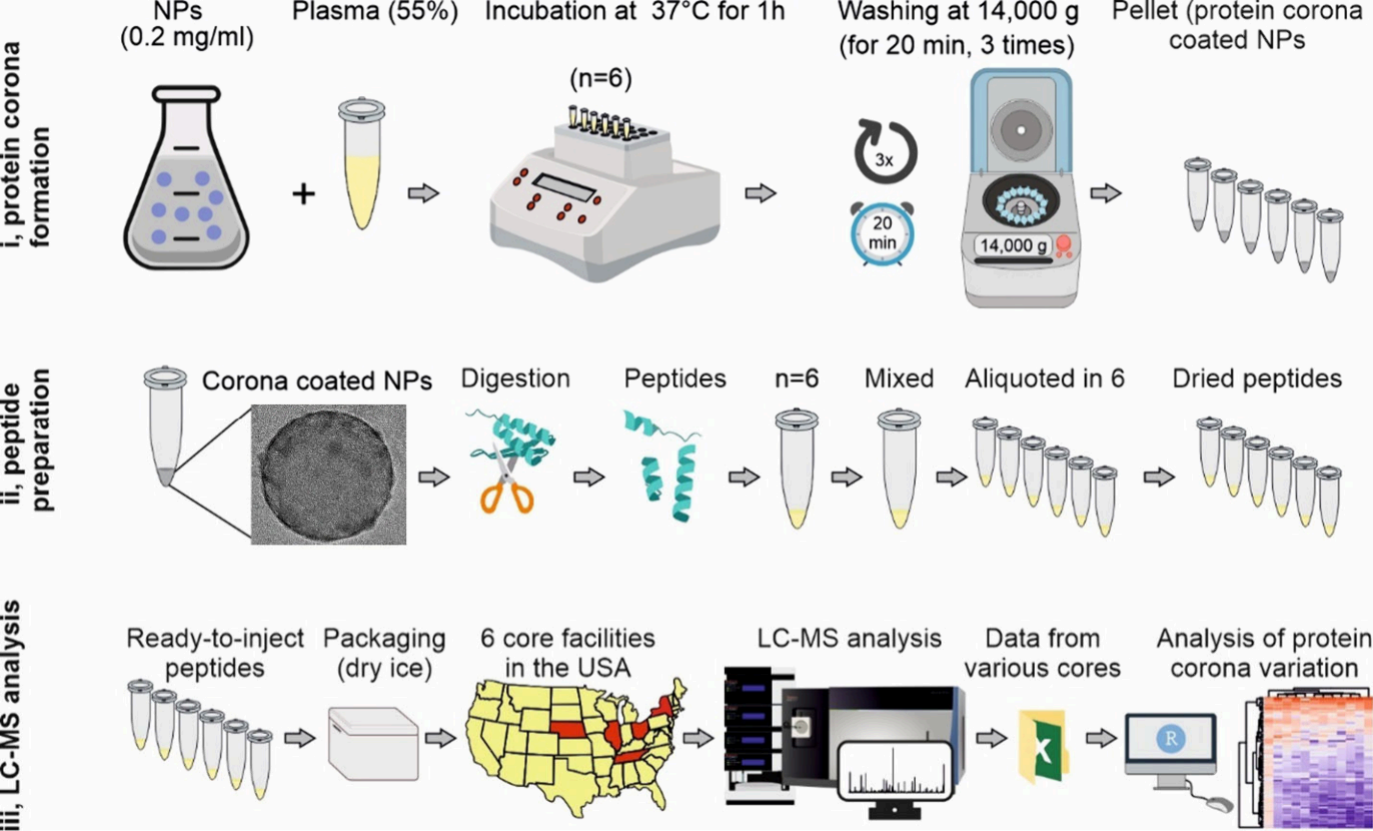
Schematic showing overall workflow of the study. After formation of 6 similar batches of protein corona–coated polystyrene nanoparticles, each individual batch was fully digested to peptides using our in-house developed protocol, mixed, aliquoted, and dried. The resulting identical aliquots were shipped to 6 different proteomics core facilities across the USA (one state includes two proteomics center) to investigate the homogeneity/heterogeneity of the protein corona composition on the surface of the nanoparticles. Out of these 6 core facilities, 2 were designated as both good and similar. The cryo-TEM image of the corona-coated nanoparticle is reproduced here with permission from reference (13).

The overall workflow of this study is illustrated in Figure 1. We employed commercially available plain polystyrene nanoparticles with an average diameter of 78.8 nm.^13^ Consistent with our previously published protocols, all protein corona-coated nanoparticles were prepared under identical conditions (refer to reference (13) for details). Supplementary Figure 1 illustrates the dynamic light scattering (DLS), zeta potential, and transmission electron microscopy (TEM) analyses of both bare and protein corona-coated nanoparticles. Notably, our prior research indicated minimal batch-to-batch variation in the physicochemical properties of the protein corona-coated nanoparticles. Bare nanoparticles were monodispersed with a narrow size distribution, measuring an average size of 78.8 ± 0.0 nm and a surface charge of −30.8 ± 0.8 mV. After exposure to human plasma, the average size increased to 111.3 ± 9.6 nm, and the surface charge changed to −10.2 ± 0.4 mV, confirming the formation of the protein corona. Detailed TEM analysis further elucidated the changes in size and morphology before and after protein corona formation, showing a distinct dark layer indicating the presence of the protein layer on the nanoparticles (Supplementary Figure 1e).^22−24^ The protein concentration in each batch, approximately 1.6 μg, was quantified using a bicinchoninic acid assay (BCA) to ensure proper sample preparation for LC-MS analysis. These fully characterized samples were subsequently digested using a standardized protocol as described in the experimental section and dispatched to various proteomics facilities for analysis.

### The Impact of Unifying Sample Preparation Protocol and Instrumentation

We submitted 4 identical batches of dried peptides to 4 core facilities that had better performance in terms of protein counts, peptide counts, coefficients of variation (CV) of technical replicates, as well as the median sequence coverage based on our previous findings^13^ (see Supporting Information for details regarding each core facility and the associated protocols). Furthermore, 4 identical batches were submitted to the 4 proteomics core facilities in the USA with similar instrumentation (with regards to LC and MS types).^13^ These centers used the same LC system (Dionex Ultimate 3000) and MS systems that should largely produce similar results (two used Fusion Lumos, one Fusion system, and one HF-X system). All centers were asked to analyze the samples over a 120 min gradient using label-free quantification (LFQ). Since good performance in our original study was also taken into account,^13^ we could not identify 4 core facilities with exact same MS instruments and had to select these centers for analysis. The LC type and MS instruments, as well as good/similar designations, are summarized in Table 1.

**Table 1:** Specific LC and MS Systems Employed by Each of the Six Core Facilities Involved in Our Study

Center blinded number	LC system	MS system	Type
1	Dionex Ultimate 3000	Fusion Lumos	Similar
2	Dionex Ultimate 3000	Eclipse	Good
3	Dionex Ultimate 3000	Fusion	Good and similar
9	Dionex Ultimate 3000	Fusion Lumos	Good and similar
11	Dionex Ultimate 3000	Eclipse	Good
13	Dionex Ultimate 3000	HF-X	Similar

**Figure 2:**
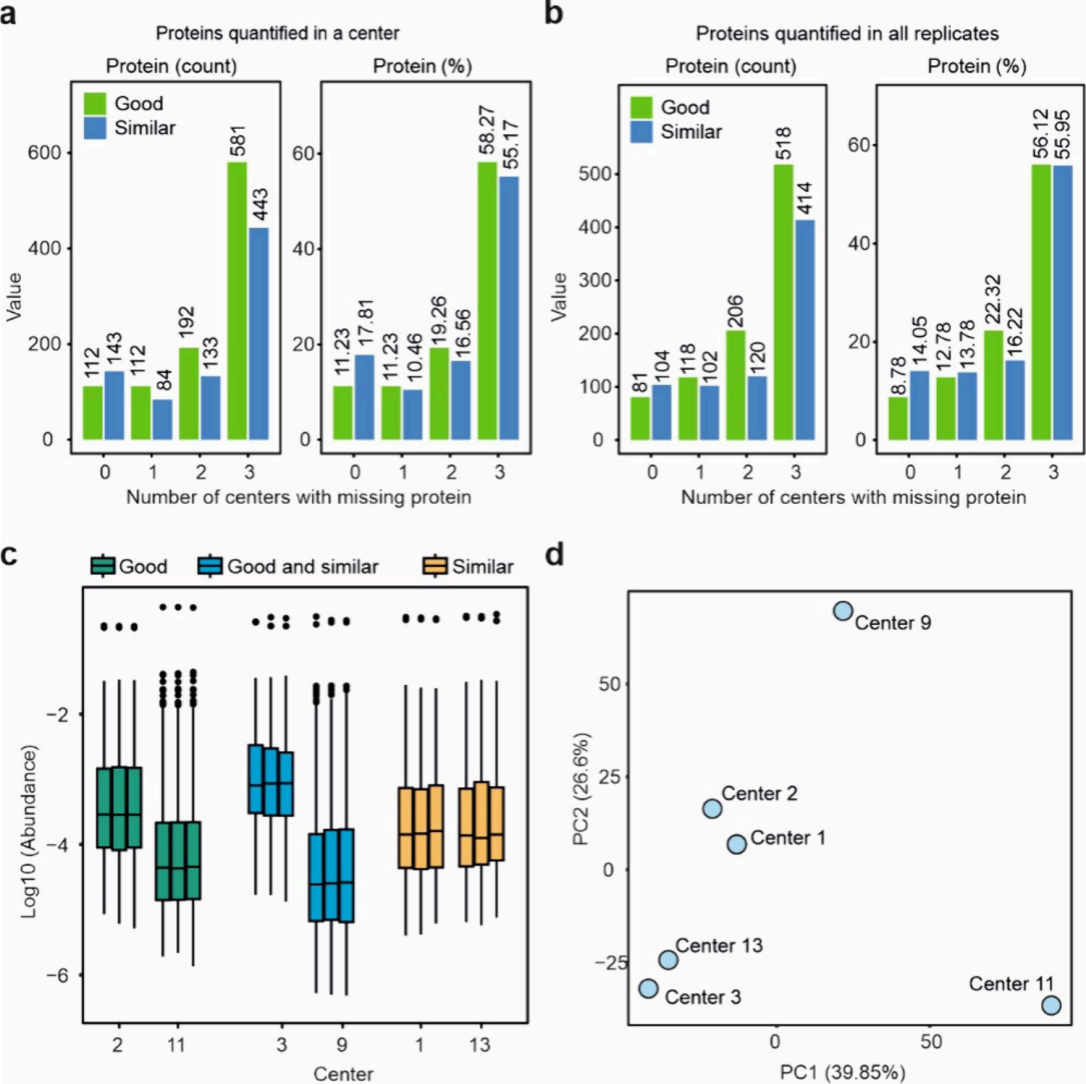
Data overlaps among core facilities reporting better results (good) or having similar instrumentation (similar). a, The overlap in the quantified proteins among good or similar centers with regards to protein count and percentage. b, The overlap in the quantified proteins among good or similar centers with regards to protein count and percentage, limited to proteins quantified in all replicates. c, Distribution of protein-level intensities for the 6 cores (center line, median; box limits contain 50%; upper and lower quartiles, 75 and 25%; maximum, greatest value excluding outliers; minimum, least value excluding outliers; outliers, more than 1.5 times upper and lower quartiles). d, PCA analysis for the proteomics data collected from the centers. All analyses were based on data from three technical replicates.

We consolidated the data from 6 core facilities as detailed in Supplementary Data 1. As depicted in Figure 2a-b, even when selecting core facilities that outperformed others based on previously mentioned criteria, the overlap of quantified proteins in identical samples across different centers was only about 11%. In contrast, for core facilities using similar instrumentation, the overlap was 18%. When considering proteins quantified consistently across all three replicates, the overlap percentages were 8% and 14% for good and similar centers, respectively. These findings highlight the impact of instrumentation on the variability of the protein corona analysis outcomes. Moreover, protein intensities between different core facilities did not exhibit a consistent pattern, regardless of whether the centers were categorized as “good” and/or “similar” (Figure 2c). A principal component analysis of the proteomics data collected from the centers is shown in Figure 2d.

**Figure 3:**
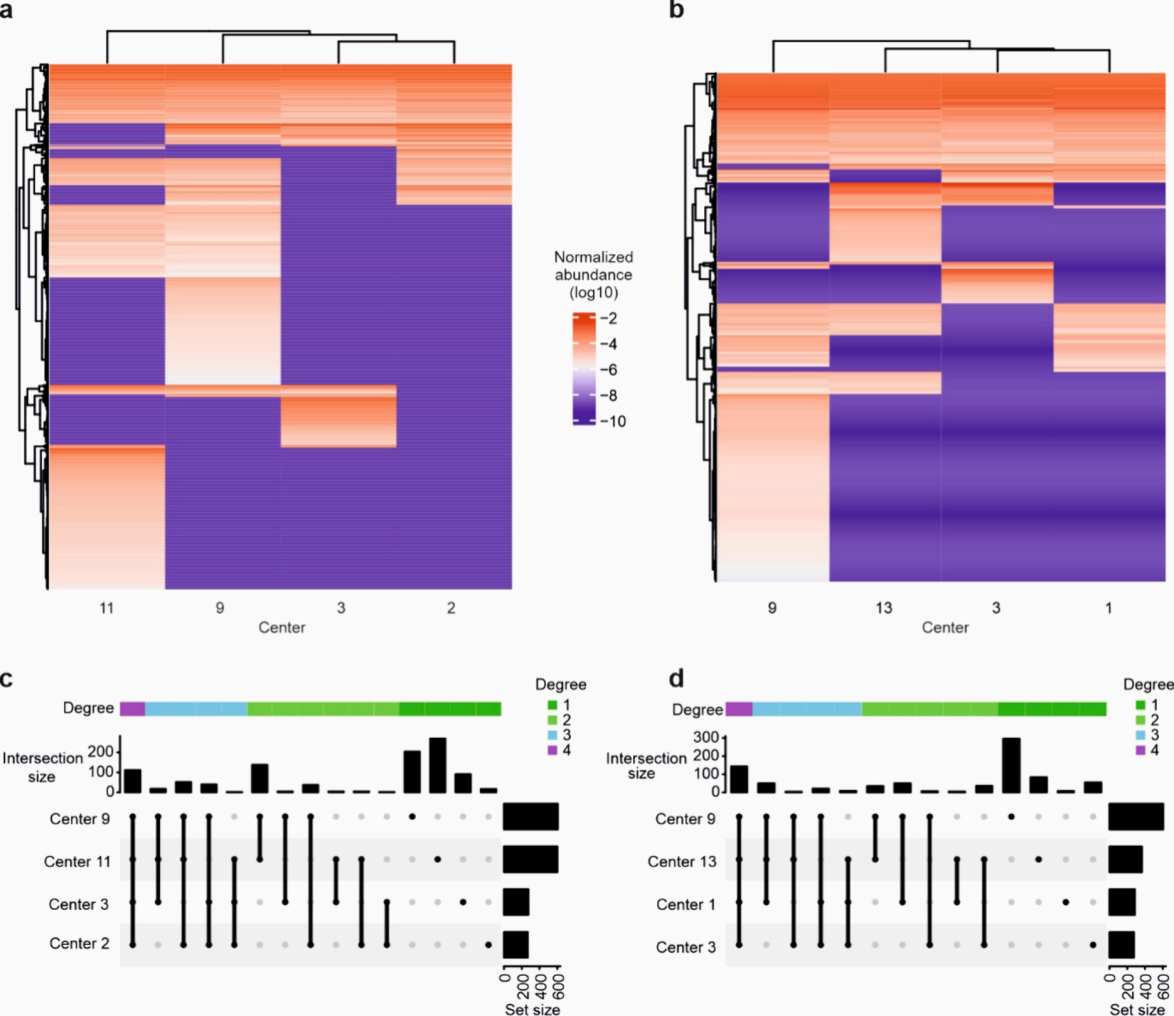
Uniqueness of the data obtained from the different core facilities. a-b, Hierarchical clustering of quantified proteins across the 4 good core facilities and 4 core facilities with similar instrumentation, respectively. c-d, Upset plot showing the variabilities in the number of detected proteins across the 4 good core facilities and 4 core facilities with similar instrumentation, respectively. All analyses were based on three technical replicates.

The hierarchical clustering of the normalized protein intensities in Figure 3a-b shows data completeness captured by each core facility for good and similar centers, respectively. The upset plots shown in Figure 3c-d demonstrate the uniqueness of the data obtained from the different core facilities for good and similar centers, respectively. These plots were made with all the proteins quantified across all the cores and demonstrate that the difference (uniqueness) of the data from each core still outweighs their similarities, despite streamlining sample preparation and the instrumentation used. Note that similarities are higher for centers using similar instruments.

### The Impact of Unifying Sample Preparation Protocol, Instrumentation, Database Search and Data Processing

As a final step, we subjected the raw data from the 4 centers with similar instrumentation to a unified database search. We have already shown that on top of the sample preparation protocol and the instruments used during sample analysis, the database search adds a new layer of variability. These variabilities can be introduced by using different search settings such as false-discovery rates (FDR), inclusion of different fixed and variable post-translational modifications, the number of missed cleavages, and the sequence database used in the search.

We included carbamidomethylation as a variable modification, since some centers had not specified if the reduction and alkylation of proteins had been performed. Carbamidomethylated peptides were used in the quantification of proteins, as well. As for other variable modifications, we included only the default methionine oxidation and acetylation of protein N-termini. Similar to previous studies by us^15^ and others,^25^ we included a 1% FDR at both the protein and peptide levels. We only searched for specific tryptic peptides and allowed up to two missed cleavages, which is a routine procedure. We only applied the parameters that are well-accepted in the community;^26^ however, it should be noted that this uniform search does not undermine the validity of the previous database searches performed by the core facilities individually.

In the uniform search, we quantified 370 proteins (Supplementary Data 2), which is significantly lower than the combined number of proteins (*n* = 1824) individually reported by the centers. A centralized FDR control and the application of the identical database search and software is expected to reduce the number of quantified proteins in the aggregated search, as we have also reported.^15^

**Figure 4:**
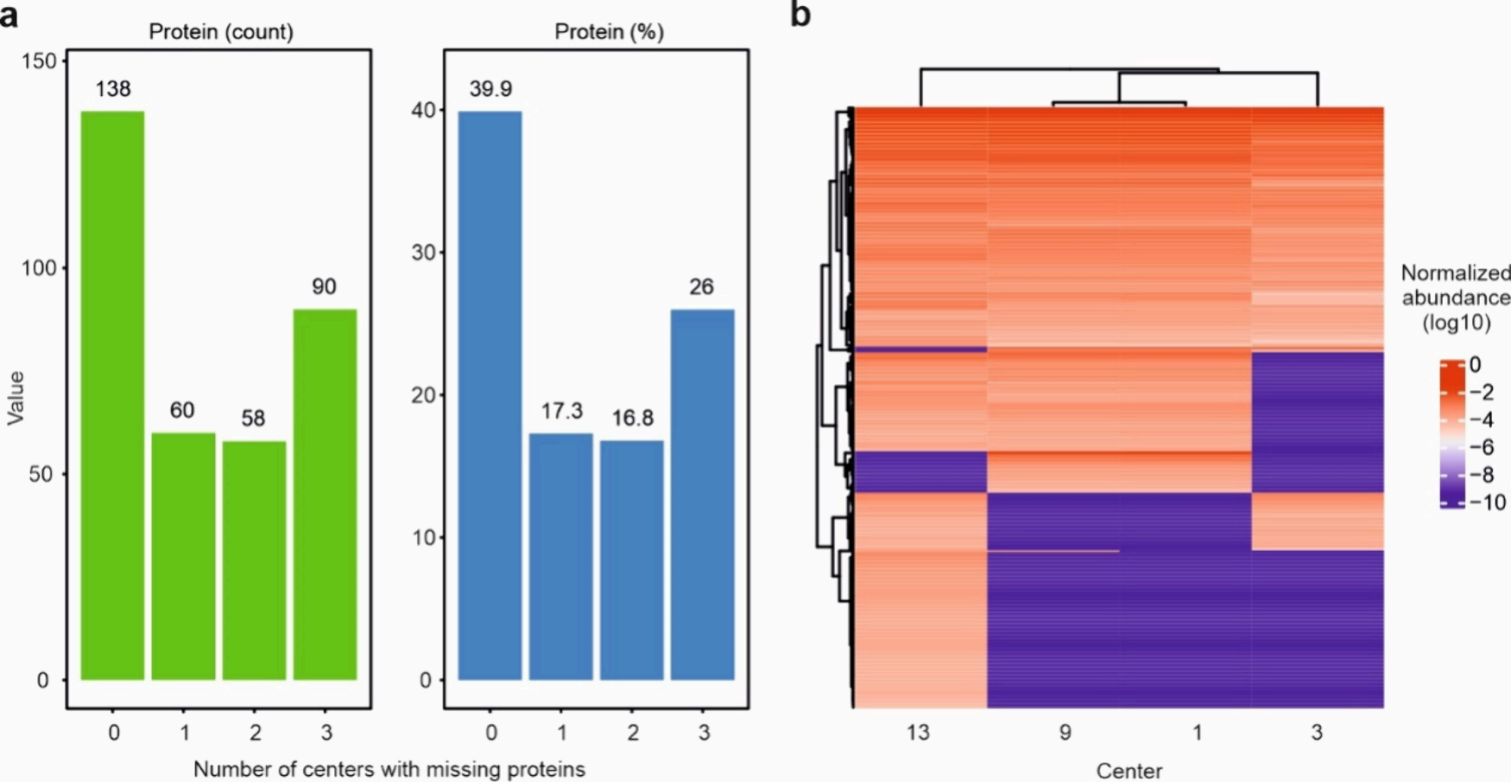
A uniform database search dramatically improves data homogeneity across different core facilities. a, The number and percentage of shared proteins with or without missing values in the uniform database search of LC-MS/MS data of the 4 centers using similar instruments, respectively. b, Proteins detected in the uniform database search of LC-MS/MS data of the 4 centers using similar instruments. All analyses were based on three technical replicates.

As shown in Figure 4a, the application of the uniform search substantially improved the number and percentage of the shared proteins in the data retrieved from the 4 core facilities using similar instrumentation. However, due to the reduction of the number of quantified proteins in the uniform database search, the percentage of shared proteins is a more accurate way of comparing performance between the individual searches and the uniform search than the number of quantified proteins. For proteins with no missing values across centers using similar instruments, the percentage of shared proteins increased from 18% in the individual searches to 40% in the uniform search. These results are consistent with our previous study, where using a uniform database search, we found an overlap of 35.3% among the top 5 facilities (among 15 centers).^15^ This is a remarkable increase, given that the MS instruments were not exactly the same and were chosen based on their expected similar performance. The hierarchical clustering in Figure 4b also shows that the data completeness across different centers was dramatically improved (compared with Figure 3b). Overall, these results show that the database search has a higher impact on the data homogeneity than the other parameters that were investigated in this study.

## Discussion

The protein corona spontaneously forms and evolves around nanoparticles when exposed to biological tissues and fluids. Recognizing its critical role in influencing the efficacy and safety of nanotechnologies and nanomedicines, extensive research has been dedicated to characterizing the protein corona composition in terms of protein identity and abundance.^14^ Despite numerous studies documented in the literature, efforts to reconcile data from independent studies and consolidate protein corona data sets for predicting nanoparticle’ pharmacokinetics and biological fates are still limited.^27^ While significant progress has been made in standardizing essential characterization methods for nanomedicines to ensure reproducibility and robustness across different research centers,^28^ a standardized protocol for analyzing the protein corona composition remains lacking. This gap highlights a critical need in the field of nanomedicine, as the protein corona plays a pivotal role in determining the biological identity and behavior of nanoparticles.^6^

MS-based proteomics is the preferred method for characterizing the protein corona. While LC-MS typically offers robust and reproducible data on cell and tissue samples within the consistent experimental framework,^29^ its application to protein corona and plasma-related samples often encounters challenges that restrict proteome coverage across different studies. A significant challenge is the broad dynamic range of protein concentrations in plasma. For example, albumin alone constitutes approximately 55% of the total protein mass in plasma. This dominance by a few high-abundance proteins can mask the presence of lower-abundance proteins, which are crucial for comprehensive proteomic analysis and accurate characterization of the protein corona.^30^ In fact, seven most abundant plasma proteins comprise 85% of total protein in plasma,^30^ and upon digestion of plasma proteome, peptides from such abundant proteins crowd the mass spectra, making it challenging to quantify the other present proteins with lower abundance. This issue has been partially mitigated in the past by several depletion strategies that exploit immunodepletion spin columns, immunodepletion-LC, magnetic beads, and even nanoparticles themselves.^10,31,32^ Such strategies are used to deplete albumin and other abundant proteins before sample analysis. We have recently also introduced a novel methodology where spiking a fine-tuned concentration of phosphatidylcholine and a single nanoparticle were shown to deplete the 4 most abundant proteins in plasma, reducing their cumulative representation (MS intensities) from 90% to under 17% in the whole plasma^33^ and enhancing the plasma proteome coverage by 446% (from 322 to 1436 plasma proteins). Despite these challenges, thousands of proteins have been reliably quantified in plasma in landmark studies, leading to the discovery of distinct disease-based biomarkers.^34−36^

Altogether, variations in proteome coverage introduce bias in data interpretation by neglecting low-abundance proteins that could otherwise be genuine targets or disease biomarkers. The need for comprehensive coverage of the proteome is felt even more in the analysis of nanoparticle protein corona, since the presence and absence of a biomarker are even more important.

Previously we showed that there are significant variations in the proteome coverage of identical protein corona samples across 17 core facilities. However, there was a good agreement in LC-MS data among the shared proteins across different core facilities, showing that one of the main challenges is achieving a high proteome coverage.^13^ We showed that these variations arise from using different sample preparation protocols, various LC-MS workflows and instruments, as well as database search parameters and data processing.^13^ In a follow-up study, we showed that implementing a uniform database search and data processing pipeline, can drastically contribute to data homogeneity (i.e., from 1.8% to 16.2% among top 11 facilities).^15^

In this study, we demonstrated that standardizing the sample preparation protocol, instrumentation, database search, and data processing significantly increases the percentage of shared proteins identified across different facilities in a stepwise manner. Our findings confirm that establishing standard protocols for LC-MS analysis of the protein corona can greatly enhance the consistency of proteomics data from various centers. Without such standardized protocols, it is challenging, if not impossible, to expect different core facilities to adopt identical settings for each step of the process. This difficulty arises from the diversity of available analytical tools, such as various types of analytical columns and database search engines. Hypothetically, homogeneity can be further improved by unifying all the instrumental settings, for example, including the duration of the gradient, fragmentation techniques, number of scans, resolution, and so on. Standardizing these elements is crucial for improving reproducibility and reliability in nanoparticle protein corona research, but will remain a formidable challenge.

In summary, the results of this study demonstrated the significant impact of unified protocols on reducing heterogeneity in identical protein corona characterization across different proteomics facilities. By implementing standardized sample preparation, using consistent instrumentation and harmonizing data processing parameters, we achieved a significant improvement in the homogeneity of the final protein corona outcomes. This highlights the critical role of standardized practices in enhancing data reproducibility and emphasizes the potential barriers in their implementation due to the diverse capabilities and resources of different laboratories. Despite these challenges, our approach illustrates the possibility of significant improvements in data quality and consistency, paving the way for achieving more reliable protein corona analysis. Within a given experiment, any of the core facilities can be used for monitoring the dynamic structure of the protein corona. The problem arises when results from multiple experiments are analyzed simultaneously, where the missing values become a problem. We recommend using a core facility that provides the highest proteome and sequence coverage (fewer missing values) and a decent CV. We further recommend designing the comparative experiments in one batch, or at least using the same protocols, workflows, and instruments when designing multibatch experiments.

Moving forward, the establishment of universal standards for the analysis of the protein corona will be crucial for the advancement of nanomedicine, ensuring that findings from different studies are comparable and that the biological implications of nanoparticle–protein interactions are understood with greater clarity. The adoption of such standards promises to bridge the gap between nanoparticle research and clinical applications, ultimately enhancing the development of nanomedicine-based diagnostics and therapeutics. It is noteworthy that this standardized protocol can be applied to both soft and hard coronas, regardless of the collection methods used. The critical factor is ensuring that the methods employed prevent any protein contamination in the protein corona shell, as contamination can lead to significant errors in data interpretation.^37^

## Supporting Information Available

Materials and methods; characterization of nanoparticles; data availability; and data analysis (PDF)
Supplementary Data 1. The consolidated data on identifcal protein corona composition from the 6 proteomics core facilities (XLSX)
Supplementary Data 2. Proteins quantified in the uniform search for the 4 core facilities using similar instruments (XLSX)

## References

[ref1] Monopoli M. P.; et al. Physical-Chemical aspects of protein corona: Relevance to in vitro and in vivo biological impacts of nanoparticles. J. Am. Chem. Soc. 2011, 133, 2525–2534. 10.1021/ja107583h. 21288025

[ref2] Shang X.; et al. Unusual zymogen activation patterns in the protein corona of Ca-zeolites. Nature Catalysis 2021, 4, 607–614. 10.1038/s41929-021-00654-6.

[ref3] Dawson K. A.; Yan Y. Current understanding of biological identity at the nanoscale and future prospects. Nat. Nanotechnol. 2021, 16, 229–242. 10.1038/s41565-021-00860-0. 33597736

[ref4] Mahmoudi M.; Landry M. P.; Moore A.; Coreas R. The protein corona from nanomedicine to environmental science. Nature Reviews Materials 2023, 8, 422–438. 10.1038/s41578-023-00552-2. PMC10037407 37361608

[ref5] Monopoli M. P.; Åberg C.; Salvati A.; Dawson K. A. Biomolecular coronas provide the biological identity of nanosized materials. Nat. Nanotechnol. 2012, 7, 779–786. 10.1038/nnano.2012.207. 23212421

[ref6] Faria M.; et al. Minimum information reporting in bio-nano experimental literature. Nat. Nanotechnol. 2018, 13, 777–785. 10.1038/s41565-018-0246-4. 30190620 PMC6150419

[ref7] Wheeler K. E.; et al. Environmental dimensions of the protein corona. Nat. Nanotechnol. 2021, 16, 617–629. 10.1038/s41565-021-00924-1. 34117462

[ref8] Leong H. S.; et al. On the issue of transparency and reproducibility in nanomedicine. Nature Nanotechnol. 2019, 14, 629–635. 10.1038/s41565-019-0496-9. 31270452 PMC6939883

[ref9] Richardson J. J.; Caruso F. Nanomedicine toward 2040.). ACS Publications (2020). 10.1021/acs.nanolett.0c00620 32118443

[ref10] Ignjatovic V.; et al. Mass spectrometry-based plasma proteomics: considerations from sample collection to achieving translational data. J. Proteome Res. 2019, 18, 4085–4097. 10.1021/acs.jproteome.9b00503. 31573204 PMC6898750

[ref11] Lan J.; et al. Systematic evaluation of the use of human plasma and serum for mass-spectrometry-based shotgun proteomics. J. Proteome Res. 2018, 17, 1426–1435. 10.1021/acs.jproteome.7b00788. 29451788

[ref12] Zimmerman L. J.; Li M.; Yarbrough W. G.; Slebos R. J.; Liebler D. C. Global stability of plasma proteomes for mass spectrometry-based analyses. Molecular & Cellular Proteomics 2012, 11, M111.014340 10.1074/mcp.M111.014340. PMC3433892 22301387

[ref13] Ashkarran A. A.; Gharibi H.; Voke E.; Landry M. P.; Saei A. A.; Mahmoudi M. Measurements of heterogeneity in proteomics analysis of the nanoparticle protein corona across core facilities. Nat. Commun. 2022, 13, 6610 10.1038/s41467-022-34438-8. 36329043 PMC9633814

[ref14] Saei A. A.; Mahmoudi M. Multi-omics exploration of biomolecular corona in nanomedicine therapeutics and diagnostics. Nanomedicine, in press , 2024, pp 1–4. 10.2217/nnm-2024-0104. PMC11285268 38593028

[ref15] Gharibi H.; Ashkarran A. A.; Jafari M.; Voke E.; Landry M. P.; Saei A. A.; Mahmoudi M. A uniform data processing pipeline enables harmonized nanoparticle protein corona analysis across proteomics core facilities. Nat. Commun. 2024, 15, 342 10.1038/s41467-023-44678-x. 38184668 PMC10771434

[ref16] Aresta A.; et al. Impact of sample preparation in peptide/protein profiling in human serum by MALDI-TOF mass spectrometry. J. Pharm. Biomed. Anal. 2008, 46, 157–164. 10.1016/j.jpba.2007.10.015. 18035512

[ref17] Ferreiro-Vera C.; Priego-Capote F.; Luque de Castro M. D. Comparison of sample preparation approaches for phospholipids profiling in human serum by liquid chromatography-tandem mass spectrometry. Journal of Chromatography A 2012, 1240, 21–28. 10.1016/j.chroma.2012.03.074. 22503623

[ref18] Klont F.; et al. Assessment of Sample Preparation Bias in Mass Spectrometry-Based Proteomics. Anal. Chem. 2018, 90, 5405–5413. 10.1021/acs.analchem.8b00600. 29608294 PMC5906755

[ref19] Vita M.; Skansen P.; Hassan M.; Abdel-Rehim M. Development and validation of a liquid chromatography and tandem mass spectrometry method for determination of roscovitine in plasma and urine samples utilizing on-line sample preparation. Journal of Chromatography B: Analytical Technologies in the Biomedical and Life Sciences 2005, 817, 303–307. 10.1016/j.jchromb.2004.12.022. 15686999

[ref20] Vuckovic D. Current trends and challenges in sample preparation for global metabolomics using liquid chromatography-mass spectrometry. Anal. Bioanal. Chem. 2012, 403, 1523–1548. 10.1007/s00216-012-6039-y. 22576654

[ref21] Wang H.; Hanash S. Intact-protein based sample preparation strategies for proteome analysis in combination with mass spectrometry. Mass Spectrom. Rev. 2005, 24, 413–426. 10.1002/mas.20018. 15389852

[ref22] Ashkarran A. A.; Dararatana N.; Crespy D.; Caracciolo G.; Mahmoudi M. Mapping the heterogeneity of protein corona by: Ex vivo magnetic levitation. Nanoscale 2020, 12, 2374–2383. 10.1039/C9NR10367H. 31960871

[ref23] Lundqvist M.; Stigler J.; Elia G.; Lynch I.; Cedervall T.; Dawson K. A. Nanoparticle size and surface properties determine the protein corona with possible implications for biological impacts. Proc. Natl. Acad. Sci. U.S.A. 2008, 105, 14265–14270. 10.1073/pnas.0805135105. 18809927 PMC2567179

[ref24] Tenzer S.; et al. Rapid formation of plasma protein corona critically affects nanoparticle pathophysiology. Nat. Nanotechnol. 2013, 8, 772–781. 10.1038/nnano.2013.181. 24056901

[ref25] Liu Y.; et al. Multi-omic measurements of heterogeneity in HeLa cells across laboratories. Nat. Biotechnol. 2019, 37, 314–322. 10.1038/s41587-019-0037-y. 30778230

[ref26] Deutsch E. W.; et al. Human proteome project mass spectrometry data interpretation guidelines 2.1. J. Proteome Res. 2016, 15, 3961–3970. 10.1021/acs.jproteome.6b00392. 27490519 PMC5096969

[ref27] Galmarini S.; et al. Beyond Unpredictability: The Importance of Reproducibility in Understanding the Protein Corona of Nanoparticles. Bioconjugate Chem. 2018, 29, 3385–3393. 10.1021/acs.bioconjchem.8b00554. 30141619

[ref28] Sharifi S.; Reuel N.; Kallmyer N.; Sun E.; Landry M. P.; Mahmoudi M. The Issue of Reliability and Repeatability of Analytical Measurement in Industrial and Academic Nanomedicine. ACS Nano 2023, 17, 4–11. 10.1021/acsnano.2c09249. 36573831 PMC10546893

[ref29] Saei A. A., Lundin A, Lyu H, Gharibi H, Luo H, Teppo J, Zhang X, Gaetani M, Végvári Á, Holmdahl R, Gygi S. P., Zubarev R. A. Multifaceted proteome analysis at solubility, redox, and expression dimensions for target identification. bioRxiv, Sept. 1, 2023. 10.1101/2023.08.31.555796 (accessed 2024-06-06). PMC11481203 39120068

[ref30] Pernemalm M.; Sandberg A.; Zhu Y.; Boekel J.; Tamburro D.; Schwenk J. M; Bjork A.; Wahren-Herlenius M.; Amark H.; Ostenson C.-G.; Westgren M.; Lehtio J. In-depth human plasma proteome analysis captures tissue proteins and transfer of protein variants across the placenta. Elife 2019, 8, e41608 10.7554/eLife.41608. 30958262 PMC6519984

[ref31] Lee P. Y.; Osman J.; Low T. Y.; Jamal R. Plasma/serum proteomics: depletion strategies for reducing high-abundance proteins for biomarker discovery. Bioanalysis 2019, 11, 1799–1812. 10.4155/bio-2019-0145. 31617391

[ref32] Gianazza E.; Miller I.; Palazzolo L.; Parravicini C.; Eberini I. With or without you—Proteomics with or without major plasma/serum proteins. Journal of proteomics 2016, 140, 62–80. 10.1016/j.jprot.2016.04.002. 27072114

[ref33] Ashkarran A. A., Deep Plasma Proteome Profiling by Modulating Single Nanoparticle Protein Corona with Small Molecules. bioRxiv, Mar. 8, 2024. 10.1101/2024.03.06.582595.

[ref34] Schwenk J. M.; et al. The human plasma proteome draft of 2017: building on the human plasma PeptideAtlas from mass spectrometry and complementary assays. J. Proteome Res. 2017, 16, 4299–4310. 10.1021/acs.jproteome.7b00467. 28938075 PMC5864247

[ref35] Keshishian H.; et al. Multiplexed, quantitative workflow for sensitive biomarker discovery in plasma yields novel candidates for early myocardial injury. Molecular & Cellular Proteomics 2015, 14, 2375–2393. 10.1074/mcp.M114.046813. 25724909 PMC4563722

[ref36] Geyer P. E.; Kulak N. A.; Pichler G.; Holdt L. M.; Teupser D.; Mann M. Plasma proteome profiling to assess human health and disease. Cell systems 2016, 2, 185–195. 10.1016/j.cels.2016.02.015. 27135364

[ref37] Mahmoudi M. The need for improved methodology in protein corona analysis. Nat. Commun. 2022, 13, 49 10.1038/s41467-021-27643-4. 35013179 PMC8748711

