# Supplementary material for: Standardizing Protein Corona Characterization in Nanomedicine: A Multicenter Study to Enhance Reproducibility and Data Homogeneity
Source: Nano Lett. 2024 Aug 3;24(32):9874–81. doi: 10.1021/acs.nanolett.4c02076 (PMC11328176; doi:10.1021/acs.nanolett.4c02076)
Supplement: Supplementary file 1 — nl4c02076_si_001.pdf [file nl4c02076_si_001.pdf]

Standardizing protein corona characterization in nanomedicine: a multi-center study to enhance reproducibility and data homogeneity

Ali Akbar Ashkarran[#1], Hassan Gharibi[#2], Seyed Majed Modaresi[3], Amir Ata Saei[4*], and Morteza Mahmoudi[1*]

[1]Department of Radiology and Precision Health Program, Michigan State University, East Lansing, MI, USA

[2]Division of Physiological Chemistry I, Department of Medical Biochemistry and Biophysics, Karolinska Institutet, Stockholm, Sweden

[3]Biozentrum, University of Basel, 4056 Basel, Switzerland

[4]Department of Microbiology, Tumor and Cell Biology, Karolinska Institutet, Stockholm 17165, Sweden

[#]Equal contribution

*Corresponding authors: (A.A.S.) amir.saei@ki.se and (M.M.) mahmou22@msu.edu

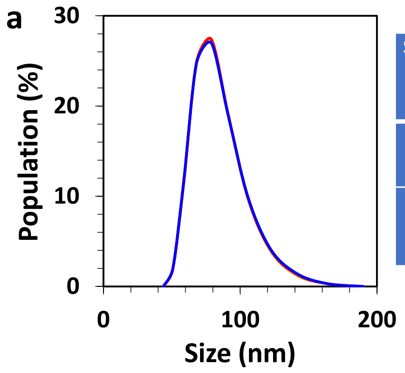

Sample	Size (nm)	SD (nm)	PDI	Zeta potential (mv)	SD (mv)
Bare NPs	78.8	0	0.028	-30.8	0.8
PC coated NPs	111.3	9.6	0.219	-10.2	0.4

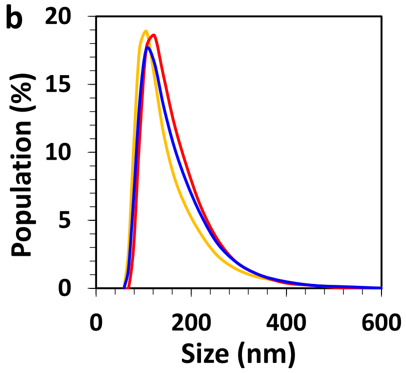

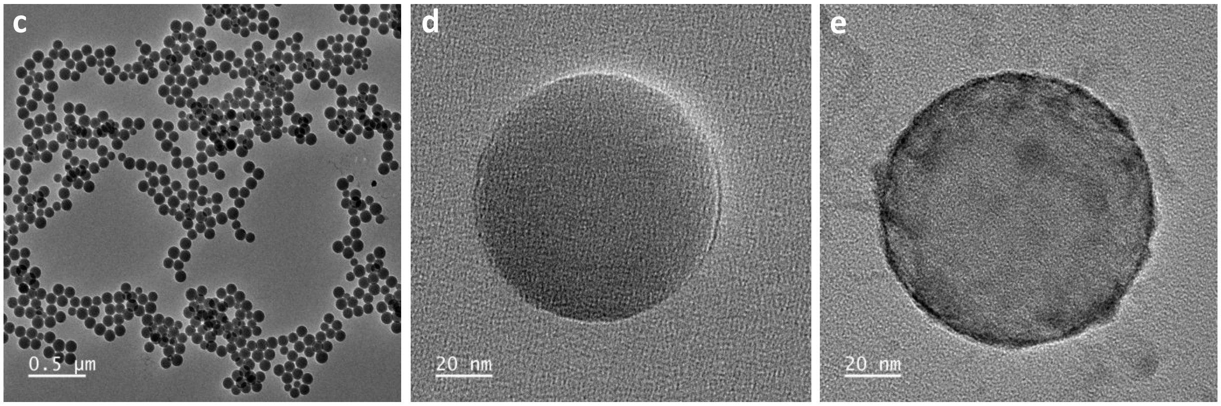

Supplementary Fig. 1. Characterizations of the pristine and protein corona-coated nanoparticles. a, and **b**, DLS analysis of pristine and protein corona–coated nanoparticles (respectively) and the corresponding replicates. Representative TEM images of nanoparticles before (**c**, and **d**) and after (**e**) formation of protein corona. The table presents the summarized average size, surface charge and polydispersity index (PDI) of pristine and protein corona-coated nanoparticles, as representative. (PC: protein corona)

Materials and Methods

Materials. Healthy human plasma protein was obtained from Innovative Research (www.innov-research.com) and diluted to a final concentration of 55% using phosphate buffer solution (PBS, 1X). Plain polystyrene nanoparticles (~ 80 nm) were provided by Polysciences. (www.polysciences.com).

Protein corona formation on the surface of nanoparticles. For protein corona formation, nanoparticles were incubated with 55% plasma (with nanoparticles' concentration of 0.2 mg/ml) for 1h at 37 °C at a constant agitation (total volume: 9´1.5 mL Eppendorf tubes). To remove unbound and plasma proteins only loosely attached to the surface of nanoparticles, protein-nanoparticles complexes were then centrifuged at 14,000 ´ g for 20 minutes, the collected nanoparticles' pellets were washed twice more with cold PBS under the same conditions, and the final pellet was collected for preparation for LC-MS analysis.

LC-MS/MS sample preparation. The protein corona pellets (i.e., 6 separate batches) obtained in the previous step were mixed with 25 µL buffer containing 50 mM ammonium bicarbonate (AMBIC) and 1.6 M Urea. Then 2.5 µL of dithiothreitol (DTT, Pierce BondBreaker, neutral pH) was added to 10 mM final concentration and incubated at 37 ^{o}C for 60 minutes using a Thermomixer. Samples were then cooled down to room temp, vortexed, and spun down. 2.7 µL of freshly made iodoacetamide (IAA) was then added to a 50 mM final concentration and samples incubated for 1 h at room temperature in dark. 3 µL of DTT was added to a 10 mM final concentration and incubated at room temperature for 15 minutes to quench the IAA. Then freshly diluted trypsin dissolved in protein digestion buffer (from Promega), was added to the samples at a ratio of 1:50 of trypsin:protein and incubated overnight at 37 ^{o}C in a Thermomixer. The following day, samples were removed from Thermomixer and cooled down to room temperature. Samples were then centrifuged at 16,000 ´ g for 20 minutes to remove the nanoparticles from supernatant. All supernatants (peptides) were transferred to separate new low binding Eppendorf tubes followed by adding 5% formic acid (FA) to the samples to adjust the pH between 2 and 3. The peptides were then desalted and cleaned using c18 StageTips in new low binding Eppendorf tubes. It is noteworthy that the c18 StageTips were first initiated using 80% acetonitrile (ACN) and 5% formic acid (FA) and equilibrated with 5% FA followed by loading and washing the sample with 5% FA. The peptides were eluted sequentially first using 30% ACN and 5% FA solution, followed by 80% ACN and 5% FA. Finally, all 6 identical eluates of prepared peptides were mixed, shaken, and aliquoted again to 6 identical batches in new low binding Eppendorf tubes and dried in a speed vacuum centrifuge. The dried peptides were then shipped overnight (using FEDEX) in dry ice with guaranteed next-morning delivery. It is noteworthy that safe arrival of all samples was confirmed by each core facility on the next day.

Characterization. DLS and zeta potential analyses were performed to measure the size distribution and surface charge of the nanoparticles before and after protein corona formation using a Zetasizer nano series DLS instrument (Malvern company). A Helium Neon laser with a wavelength of 632 nm was used for size distribution measurement at room temperature. TEM was carried out using a JEM-2200FS (JEOL Ltd.) operated at 200kV. The instrument was equipped with an in-column energy filter and an Oxford X-ray energy dispersive spectroscopy (EDS) system. 20 µl of the bare nanoparticles were deposited onto a copper grid and used for imaging. For protein corona–coated nanoparticles, 20 µL of sample was negatively stained using 20 µl uranyl acetate 1%, washed with DI water, deposited onto a copper grid, and used for imaging. Protein corona composition was also determined using LC-MS/MS. LC-MS/MS analyses were carried out at 6 different proteomics cores across the United States that performed better based on our previous findings and had similar instrumentation (i.e., LC and mass types).

BCA analysis

To estimate the concentration of the proteins in identical batches of protein corona-coated nanoparticles, we measured the protein concentration using bicinchoninic acid assay (BCA). BCA analysis confirmed the consistency of protein amount in the identical batches of protein corona coated nanoparticles. In the low concentrations of BSA, where there is a meaningful linear relationship between the concentrations and absorption values, the protein concentration was calculated about ~ 1.6 µg in each batch after protein corona formation. In general, there is 20-40% loss in protein concentration during digestion and sample preparations for LC-MS analysis and therefore, we estimate that each vial contains less than 1 µg protein. Since we asked all proteomics core to resuspend and split the sample into three technical replicates, and therefore, in each individual analysis ~ 350 ng protein was analyzed by LC-MS system. It is noteworthy that none of the cores reported any issues such as column clogging during the measurements.

LC-MS

The following sections describe the experimental details and instrumentation of LC-MS/MS analysis provided by each individual proteomic core facility and reported here as received (with some minor changes for consistency of units, etc.). The 6 different proteomics core facilities that contributed to this study include Cornell University (Link), Cleveland Clinic Lerner Research Institute (Link), University of Cincinnati (Link), University of Tennessee (Link), University of Nebraska–Lincoln (Link), University of Illinois (Link). The centers were blindly numbered as 1, 2, 3, 9, 11, and 13 in random order corresponding to our previous report.[1]

Center #1:

The tryptic digest sample was reconstituted in 30 µL of 1% acetonitrile (ACN) with 0.5% formic acid (FA) for nanoLC-ESI-MS/MS analysis and aliquoted to three separate sample vials. The analysis was carried out using a Dionex Ultimate 3000 nano-flow HPLC connected to an Orbitrap FusionTM TribridTM (Thermo-Fisher Scientific, San Jose, CA) mass spectrometer equipped with a nanospray Flex Ion Source, and coupled with a Dionex UltiMate 3000 RSLCnano system (Thermo, Sunnyvale, CA). The peptide samples (5 µL) of each aliquot were injected onto a PepMap C-18 RP nano trapping column (5 µm, 100 µm i.d. x 20 mm) at 5 µL/min flow rate for rapid sample loading and then separated on a PepMap C-18 RP nano column (2 µm, 75 µm i.d. x 25 cm) at 35°C. The tryptic peptides were eluted in a 105-min gradient of 2% to 35% ACN in 0.1% formic acid at 300 nL/min, followed by a 10-min ramping to 90% ACN-0.1% FA and an 7-min hold at 90% ACN-0.1% FA. The column was re-equilibrated with 0.1% FA for 11 min prior to the next run. The Orbitrap Fusion was operated in positive ion mode with spray voltage set at 2.3 kV and source temperature at 250°C. External calibration for FT, IT and quadrupole mass analyzers were performed. In data-dependent acquisition (DDA) analysis, the instrument was operated using FT mass analyzer in MS scan to select precursor ions followed by 3 second "Top Speed" data-dependent CID ion trap MS/MS scans at 1.6 m/z quadrupole isolation for precursor peptides with multiple charged ions above a threshold ion count of 5000 and a collision energy of 35%. MS survey scans at a resolving power of 120,000 (FWHM at m/z 200), for the mass range of m/z 350-1500. Dynamic exclusion parameters were set at 60 s of exclusion duration with ±1 0 ppm exclusion mass width. All data were acquired under Xcalibur 4.4 operation software (Thermo-Fisher Scientific).

The DDA raw files with MS and MS/MS were subjected to database searches using Sequest which is bundled into Proteome Discoverer version 2.5 in an LFQ workflow against *Homo sapiens*

SwissProt database containing 26,576 sequences along. Oxidation on M, acetylation of protein N-termini, and deamidation on N were specified as dynamic modifications, while carbamidomethylation on C was specified as a static modification, and a maximum of 2 missed cleavages by trypsin digestion was allowed. The peptide mass tolerance in MS mode was set to 10 ppm and MS/MS tolerance was set to 0.6 Da. The estimated false discovery rate (FDR) thresholds for protein, peptide and modification site were specified at a maximum of 1%. The minimum peptide length was set at 6 and unique and razor peptide intensities were used. All the other parameters in PD 2.5 were set to default values.

Relative quantitation of identified proteins between the three technical replicates was determined by using and LFQ approach and the chromatographic alignment and featuer detection was performed using the Minora node low in PD 2.5. The precursor abundance intensity (the output of the Minora node, for each peptide identified by MS/MS in each replicate was automatically determined and their unique plus razor peptides for each protein in each replicate were summed and used for calculating the protein abundance.

Center #2:

The dried sample was resuspended in 30 µL of 0.1% formic acid/2% acetonitrile and 5 µL of the sample was injected in triplicate onto a 5 mm nanoviper µ-Precolumn (i.d.300 µm, C18 PepMap 100, 5.0 µm, 100 Å) from ThermoFisher Scientific at 5 µl/min in formic acid/H_2O 0.1/99.9 (v/v) for 5 min to desalt and concentrate the samples. The analysis was carried out using on an Orbitrap Eclipse mass spectrometer (ThermoFisher Scientific) coupled to a Dionex Ultimate 300 RSLCnano system (ThermoFisher Scientific). For the chromatographic separation of peptides, the trap-column was switched to align with the EASY-Spray column PepMap RSLC C18 with a 150 mm column (i.d. 75 µm, C18, 3.0 µm, 100 Å). The peptides were eluted using a variable mobile phase (MP) gradient from 98% phase A (Formic acid/H_2O 0.1/99.9, v/v) to 32% phase B (Formic Acid/Acetonitrile 0.1/99.9, v/v) for 120 min at 300 nL/min. MS1 data were collected in the Orbitrap (120,000 resolution; maximum injection time 50 ms; AGC 4×10^5). Charge states between 2 and 6 were required for MS2 analysis, and a 20 s dynamic exclusion window was used. Cycle time was set at 2.5 s. MS2 scans were performed in the ion trap with HCD fragmentation (isolation window 0.8 Da; NCE 30%; maximum injection time 40 ms; AGC 5×10^4). The data was recorded using Thermo Scientific Xcalibur 4.5 software.

The MS raw files were subjected to database searches against a combined database containing common contaminants and the *Homo sapiens* (UP000005640) database using Proteome discoverer version 2.4 with the Sequest HT search algorithm (Thermo scientific) using a multiconsensus by sample run Label Free Quantitation (LFQ) workflow which provides abundance values for each sample with ratios and still shows the Sequest scores, peptides and PSMs for each file individually.

Center #3:

The tryptic digest sample was reconstituted in 30 µL of 2% acetonitrile (ACN) with 0.5% formic acid (FA) for nanoLC-ESI-MS/MS analysis and aliquoted to three separate sample vials. The analysis was carried out using a Dionex Ultimate 3000 nano-flow HPLC connected to an Orbitrap FusionTM TribridTM (Thermo-Fisher Scientific, San Jose, CA) mass spectrometer equipped with a nanospray Flex Ion Source, and coupled with a Dionex UltiMate 3000 RSLCnano system (Thermo, Sunnyvale, CA).[2, 3] The peptide samples (5 µL) of each aliquot were injected onto a PepMap C-18 RP nano trapping column (5 µm, 100 µm i.d. x 20 mm) at 20 µL/min flow rate for

rapid sample loading and then separated on a PepMap C-18 RP nano column (2 µm, 75 µm i.d. x 25 cm) at 35°C. The tryptic peptides were eluted in a 120-min gradient of 5% to 35% ACN in 0.1% formic acid at 300 nL/min, followed by a 7-min ramping to 90% ACN-0.1% FA and an 8-min hold at 90% ACN-0.1% FA. The column was re-equilibrated with 0.1% FA for 25 min prior to the next run. The Orbitrap Fusion was operated in positive ion mode with spray voltage set at 1.1 kV and source temperature at 275°C. External calibration for FT, IT and quadrupole mass analyzers were performed. In DDA analysis, the instrument was operated using FT mass analyzer in MS scan to select precursor ions followed by 3 second "Top Speed" data-dependent CID ion trap MS/MS scans at 1.6 m/z quadrupole isolation for precursor peptides with multiple charged ions above a threshold ion count of 10,000 and normalized collision energy of 30%. MS survey scans at a resolving power of 120,000 (FWHM at m/z 200), for the mass range of m/z 375-1575. Dynamic exclusion parameters were set at 50 s of exclusion duration with ±10 ppm exclusion mass width. All data were acquired under Xcalibur 4.4 operation software (Thermo-Fisher Scientific).

The DDA raw files with MS and MS/MS were subjected to database searches using MaxQuant version 2.3.1.0 in an LFQ workflow against *Homo sapiens* Uniprot database containing 26,019 sequences along with a regular contaminant (244 entries) database. Oxidation on M, acetylation of protein N-termini, and deamidation on N were specified as dynamic modifications, while carbamidomethylation on C was specified as a static modification, and a maximum of 2 missed cleavages by trypsin digestion was allowed. The peptide mass tolerance in MS mode was set to 10 ppm and MS/MS tolerance was set to 0.6 Da. The estimated false discovery rate (FDR) thresholds for protein, peptide and modification site were specified at a maximum of 1%. The minimum peptide length was set at 6 and unique and razor peptide intensities were used. All the other parameters in MaxQuant were set to default values.[4]

Relative quantitation of identified proteins between the three technical replicates was determined by the LFQ workflow in MaxQuant. The precursor abundance intensity (the output of the MaxLFQ algorithm,[5] for each peptide identified by MS/MS in each replicate was automatically determined and their unique plus razor peptides for each protein in each replicate were summed and used for calculating the protein abundance.

Center #9:

Each dried peptide sample (~1ug) was re-dissolved in 25 µl of loading buffer (3% acetonitrile, 0.1% TFA), and 5 µl (0.2ug) was analyzed using LC-MS-MS method with 120min LC gradient for peptide/protein identification and label-free quantification (LFQ); each sample was analyzed in triplicate. Raw MS data were acquired on an Orbitrap Fusion Lumos tribrid mass spectrometer equipped with Nanospray Flex Ion Source (Thermo Fisher) and operating under Xcalibur 4.3 in line with Ultimate 3000RSLCnano UHPLS system (Thermo Fisher). The Orbitrap Fusion Lumos mass-spectrometer was operated in positive ion mode with spray voltage set at 2.0 kV and Ion Transfer Tube (ITT) temperature at 275°C. External calibration for Orbitrap, Linear Ion Trap, and quadrupole mass analyzers were performed. The peptides were trapped on an Acclaim PepMap 100 nanoViper column (75µm x 20mm, Thermo Fisher) at 5ul/min flow rate for 5min. The trapped peptides were separated on an Acclaim PepMap RSLC nanoViper column (75µm x 500mm, C-18, 2µm, 100Å, Thermo Fisher) at 300nl/min flow rate and 40 °C column temperature using water and acetonitrile with 0.1% formic acid as solvents A and B, respectively. The following multi-point linear gradient was applied for peptide elution: 3%B at 0-4min, 5%B at 5min, 25%B at 55min, 30%B at 60min, 90%B at 63-73min, and 3%B at 76-100min. DDA method was used with 3sec

cycles and the following MS scan parameters. Full, survey MS scans were performed in the Orbitrap analyzer at 120,000 resolving power (FWHM, at m/z=200) for the mass range m/z 375-1500. The following data dependent MS2 analysis was performed on precursor ions with peptide-specific isotopic pattern, charge state 2-6, and intensity of at least 50,000. For MS2 scans, peptide ions were isolated using quadrupole isolation with 0.7 m/z window, fragmented (HCD, 30% NCE), and the fragment masses were determined in the Orbitrap analyzer at 30,000 resolving power (FWHM, at m/z=200). Dynamic exclusion was applied for 30sec with ±10 ppm exclusion mass width.

The analysis the acquired raw MS data was performed within a mass informatics platform Proteome Discoverer 2.4 (Thermo Fisher) using Sequest HT search algorithm and human protein database (SwissProt, Homo sapiens, TaxID 9606, v.2023-06-28, 42370 entries). The reversed target database was used as decoy database. Full tryptic peptides were searched; 2 miss-cleavages were allowed. The searched fixed modifications included: carbamidomethylation of Cys. The variable modifications included oxidation of Met and acetylation of the protein N-terminus. The precursor and fragment ion mass tolerances were set to 10ppm and 0.02Da, respectively. The raw data were filtered for the precursor ions with S/N of at least 1.5. The PSMs were filtered for further analysis using a delta Cn threshold of 0.05. The FDR threshold of 0.01 was used to validate and filter the data at PSM (Percolator), and then at peptide (Qvality algorithm) levels. The validated/filtered peptides were used for the identification of the candidate precursor proteins. Each candidate precursor protein was scored by summing the PEP values of the assigned peptides. The sum-PEP protein scores were used to further validate (without filtering) the candidate proteins using 0.01 (strict) and 0.05 (relaxed) FDR thresholds.

Label free quantitation (LFQ) was used for sample proteins identified in three technical replicates. The following parameters were used for feature detection, chromatographic alignment, and feature linking: 5 for minimal trace length, 10ppm for mass tolerance, automatic for RT tolerance, 5 for minimal S/N threshold. Peptide quantification was based on LC peak area with at least 5 data-points. Protein abundances were determined as summed abundances of assigned peptides. Unique and razor peptides were used for protein quantification.

Center #11

The tryptic digest sample was reconstituted in 30 µL of 2% acetonitrile (ACN) with 0.5% formic acid (FA) for nanoLC-ESI-MS/MS analysis and aliquoted to three separate sample vials. The analysis was carried out using an Orbitrap EclipseTM TribridTM (Thermo Fisher Scientific, San Jose, CA) mass spectrometer equipped with a nano EASY-spray and coupled with a Dionex UltiMate 3000 RSLCnano system (Thermo Fisher Scientific). The peptide samples (5 µL) of each aliquot were injected onto an Acclaim PepMap 100 trapping column (5 µm, 75 µm i.d. x 20 mm) at 5 µL/min flow rate for rapid sample loading and then separated on an Acquity UPLC M-class peptide CSH 130A (1.7 µm, 75 µm i.d. x 25 cm) C18 nano column (Waters Corp, Milford, MA) at 35°C. The tryptic peptides were eluted in a 96-min gradient of 4% to 22% ACN in 0.1% formic acid at 300 nL/min, followed by a 2-min ramping to 80% ACN-0.1% FA and a 3-min hold at 80% ACN-0.1% FA. The column was re-equilibrated with 0.1% FA for 16 min prior to the next run. The Orbitrap Eclipse was operated in positive ion mode with spray voltage set at 2.0 kV and source temperature at 305°C. External calibration for FT, IT and quadrupole mass analyzers were performed. In DDA analysis, the instrument was operated using FT mass analyzer in MS scan to select precursor ions followed by 3 second "Top Speed" data-dependent HCD ion trap MS/MS scans at 1.4 m/z quadrupole isolation for precursor peptides with multiple charged ions above a

threshold ion count of 10,000 and normalized collision energy of 30%. MS survey scans at a resolving power of 120,000 (FWHM at m/z 200), for the mass range of m/z 350-1350. Dynamic exclusion parameters were set at 60 s of exclusion duration with ±10 ppm exclusion mass width. All data were acquired under Xcalibur 4.7 operation software (Thermo Fisher Scientific).

The DDA raw files with MS and MS/MS were subjected to database searches using Proteome Discoverer (Thermo Fischer Scientific) version 2.4 in an LFQ workflow against *Homo sapiens* Uniprot database containing 80,581 (including non-canonical) sequences along with a regular contaminant (125 entries) database. Oxidation on M, and deamidation on N were specified as dynamic modifications, while carbamidomethylation on C was specified as a static modification, and a maximum of 2 missed cleavages by trypsin digestion was allowed. The peptide mass tolerance in MS mode was set to 15 ppm and MS/MS tolerance was set to 0.6 Da. The estimated false discovery rate (FDR) thresholds for protein, peptide and modification site were specified at a maximum of 1%. The minimum peptide length was set at 6 and unique and razor peptide intensities were used.

Relative quantitation of identified proteins between the three technical replicates was determined by the LFQ workflow in Proteome Discoverer. Peptides were validated by Percolator with a 0.01 posterior error probability threshold. The data were searched using a decoy database to set the false discovery rate to 1% (high confidence). The peptides were quantified using the precursor abundance based on intensity. The peak abundance was normalized using total peptide amount. Only proteins and peptides identified and quantified in at least one of the technical replicates were reported.

Center #13:

The tryptic digest sample was reconstituted in 30 µL 2% acetonitrile (ACN) with 0.1% formic acid (FA) for nanoLC-ESI-MS/MS analysis. The analysis was carried out using a Dionex UltiMate 3000 RSLCnano system (Thermo, Sunnyvale, CA) connected to a Q Exactive HF-X (Thermo-Fisher Scientific, San Jose, CA) mass spectrometer equipped with a nanospray Flex Ion Source. The peptide samples (equal amount from the same vial for each run) were separated on an Acclaim PepMap C-18 100 RP nano column (2 µm, 75 µm i.d. x 25 cm) at 50°C. The tryptic peptides were eluted in a 120-min gradient of 5% to 35% B (80% ACN in 0.1% formic acid) at 300 nL/min, followed by a 10-min ramping to 50% B, a 6-min ramping to 90% B, and a 6-min hold at 90% B. The column was re-equilibrated with 2% B for 10 min prior to the next run. The Q Exactive HF-X was operated in positive ion mode with spray voltage set at 2.0 kV and source temperature at 275°C. External calibration for FT and quadrupole mass analyzers were performed.

In DDA analysis, the instrument was operated using the FT mass analyzer in MS scan to select the top 15 most abundant precursor ions for data-dependent HCD MS/MS scans at 1.0 m/z quadrupole isolation for precursor peptides with multiple charged ions and normalized collision energy of 30%. MS survey scans at a resolving power of 120,000 (FWHM at m/z 200) were acquired for the mass range of m/z 350-1500. Dynamic exclusion parameters were set at 60 s of exclusion duration. All data were acquired under Xcalibur 4.2.47 operation software (Thermo-Fisher Scientific).

The DDA raw files with MS and MS/MS were subjected to database searches using MaxQuant version 2.0.1.0 in an LFQ workflow against *Homo sapiens* Uniprot database containing 20,516 sequences along with the standard MaxQuant common contaminant database. Oxidation on M and acetylation of protein N-termini were specified as dynamic modifications, while

carbamidomethylation on C was specified as a static modification, and a maximum of 2 missed cleavages by trypsin digestion was allowed. The peptide mass tolerance in MS mode was set to 10 ppm and MS/MS tolerance was set to 20 ppm. The estimated false discovery rate (FDR) thresholds for protein, peptide and modification site were specified at a maximum of 1%. The minimum peptide length was set at 6 and unique and razor peptide intensities were used. All the other parameters in MaxQuant were set to default values.

Relative quantitation of identified proteins between the three technical replicates was determined by the LFQ workflow in MaxQuant. The precursor abundance intensity (the output of the MaxLFQ algorithm) for each peptide identified by MS/MS in each replicate was automatically determined and their unique plus razor peptides for each protein in each replicate were summed and used for calculating the protein abundance.

Data availability

Due to the blinding of core names in the current study, and since the .raw files can be traced, the .raw data and associated individual data files are available upon request from corresponding authors. The extracted protein abundances data and relevant outputs of data analysis are provided in supplementary data files cited in the text. **Supplementary Data 1** was used to generate **Figs. 2-3** and **Supplementary Data 1** for **Fig 4** (**Supplementary Data 1-2** as the Source Data).

Data analysis

First, for each core, data were normalized by total protein intensity in each technical replicate. CVs were calculated based on normalized intensities between technical replicates for each protein. To unify protein IDs, for some cores, the various protein IDs used were converted to UniProt IDs. The data among the cores were combined by UniProt IDs.

Acknowledgements:

We thank the Proteomics and Metabolomics Facility of Cornell University for providing the mass spectrometry data and NIH SIG grant 1S10 OD017992-01 support for the Orbitrap Fusion mass spectrometer. This work was supported by the Vincent Coates Foundation Mass Spectrometry Laboratory, Stanford University Mass Spectrometry (RRID:SCR_017801). This work was supported in part by NIH P30 CA124435 utilizing the Stanford Cancer Institute Proteomics/Mass Spectrometry Shared Resource.

References

1. Ashkarran AA, Gharibi H, Voke E, Landry MP, Saei AA, Mahmoudi M. Measurements of heterogeneity in proteomics analysis of the nanoparticle protein corona across core facilities. *Nature Communications* **13**, 6610 (2022).

2. Yang Y, Anderson E, Zhang S. Evaluation of six sample preparation procedures for qualitative and quantitative proteomics analysis of milk fat globule membrane. *Electrophoresis* **39**, 2332-2339 (2018).

3. Harman RM, He MK, Zhang S, Van De Walle GR. Plasminogen activator inhibitor-1 and tenascin-C secreted by equine mesenchymal stromal cells stimulate dermal fibroblast migration in vitro and contribute to wound healing in vivo. *Cytotherapy* **20**, 1061-1076 (2018).

4. Cox J, Mann M. MaxQuant enables high peptide identification rates, individualized p.p.b.-range mass accuracies and proteome-wide protein quantification. *Nature Biotechnology* **26**, 1367-1372 (2008).

5. Cox J, Hein MY, Luber CA, Paron I, Nagaraj N, Mann M. Accurate proteome-wide label-free quantification by delayed normalization and maximal peptide ratio extraction, termed MaxLFQ. *Molecular and Cellular Proteomics* **13**, 2513-2526 (2014).
